# The PLUS study: efficacy of triclosan coated suture (VicrylPlus^®^) to reduce infection in primary suture of childbirth related perineal tears - a randomized controlled trial

**DOI:** 10.1186/s40748-025-00211-0

**Published:** 2025-05-05

**Authors:** K. Sonnichsen, P-E. Isberg, J. Elers, M. Zaigham, Nana Wiberg

**Affiliations:** 1 Department of Obstetrics and Gynecology, Skåne University Hospital, Malmö and Lund, Sweden; 2 Department of Statistics, Lund University, Lund, Sweden; 3 Department of Gynecology and Obstetrics, Holbaek Hospital, Holbaek, Denmark; 4 Obstetrics & Gynecology, Institution of Clinical Sciences Lund, Lund University, Lund, Sweden; 5 Department of Clinical Sciences Malmö, Lund University, Lund, Sweden; 6 Department of Gynecology and Obstetrics, Sjaelland University Hospital, Roskilde, Denmark

**Keywords:** Antibacterial sutures, Childbirth, Episiotomies, Perineal tears, Surgical site infection

## Abstract

**Background:**

Preventing infection in primary sutured perineal tears after childbirth is crucial to avoid systemic antibiotic use and potential complications from poor healing. This study aimed to investigate the efficacy of an antibacterial, triclosan-coated suture (VicrylPlus^®^) in reducing infection in primary sutured childbirth-related perineal tears.

**Methods:**

The PLUS study was a single-center, single-blinded, adaptive parallel-group randomized trial conducted at Lund University Hospital, Sweden. Women aged ≥ 18 years with a perineal tear at childbirth were randomly assigned in a 1:1 ratio to either the control group (conventional-absorbable suture, Vicryl^®^) or the intervention group (triclosan-coated- absorbable suture, VicrylPlus^®^).

**Results:**

Out of 1921 eligible women, 1890 were randomized to either Vicryl^®^ (*n* = 953) or VicrylPlus^®^ (*n* = 937). There were no significant demographic differences between the groups. The most common type of tear in both groups was a second-degree tear (Vicryl^®^ 66.2% (*n* = 625), VicrylPlus^®^ 67.5% (*n* = 625)). Encompassing all types of deeper tears in the analysis there was a significantly decrease in infection after suturing with VicrylPlus^®^ 4% (*n* = 28) versus Vicryl^®^ 6.8% (*n* = 47); (OR 0.57, 95% CI 0.35–0.91, *P* = 0.024). When analyzing different tears separately, there was a non-significant increase in infection for first-degree tears with VicrylPlus^®^ 0.8% (*n* = 2) versus Vicryl^®^ 3.9% (*n* = 8); (OR 4.75, 95% CI 1.00-22.63, *P* = 0.050). However, for second-degree tears, the infection rate was significantly reduced with VicrylPlus^®^ 4.4% (*n* = 27) versus Vicryl^®^ 7.2% (*n* = 44); (OR 0.63, 95% CI 0.36–0.98, *P* = 0.05) and for third-degree and unclassified tears there was a non-significant decrease in infections with VicrylPlus^®^ 5.3% (*n* = 1) versus Vicryl^®^ 14.3% (*n* = 2); (OR 0.33, 95% CI 0.03–4.10, *P* = 0.561), respectively, VicrylPlus^®^ 0% versus Vicryl^®^ 1.7% (*n* = 1); (OR 0.98, 95% CI 0.95–1.02, *P* = 0.462).

**Conclusion:**

The use of triclosan coated sutures significantly reduces the risk of infection in primary sutured childbirth-related perineal tears by 43%, except for first-degree tears. Further research is needed to determine whether their effectiveness remains consistent across the other specific types of deeper tears in a larger study population.

**Trial registration:**

ClinicalTrials (NCT02863874), posted 11/08/2016, retrospectively registered. Approved by the regional ethical committee before start of enrollment (Dnr 2015/774).

**Supplementary Information:**

The online version contains supplementary material available at 10.1186/s40748-025-00211-0.

## Background

Approximately 85% of women experience injury to the vagina, vulva, and pelvic floor muscles during childbirth, with around 70% requiring suturing after a vaginal birth [1–3]. Childbirth imposes significant physical and psychological demands and suffering a painful perineal tear eventually with physical restrictions can cause embarrassment and anxiety about future perineal health and function. These issues can potentially lead to postnatal depression or post-traumatic symptoms [4–6]. Visible perineal tears are classified into first- through fourth-degree tears, a system first proposed by Sultan in 1999 [7]. The incidence of the different types of tears are shown to be dependent on parity, assisted vaginal birth, longer duration of second stage, height of the perineum, fetal birthweight and connective tissue disorder and is reported from: first degree tears 5–12%, spontaneous second-degree tears 35–78%, episiotomy 7–31%, third degree 2.4-8%, fourth 0.2–0.3% [8–10]. The healing process of perineal tears is not well understood and despite increasing awareness and interest in pelvic floor health after birth, follow-up care for women with perineal tears has been under-prioritized politically [11]. An infection that complicates delayed or abnormal anatomical healing may contribute to pelvic floor dysfunction (PFD), a condition affecting 25–50% of women during their lifetime [12–16]. Therefore, conditions that facilitates healing of a childbirth related tear (CRPT) should be prioritized. It is often challenging to reverse pre-existing comorbidities or behavior patterns, making it essential to optimize intervention-related factors. Suture material is crucial, as it provokes an inflammatory response until it is absorbed. In the Western world, absorbable, braided, rapidly or intermittently long-lasting sutures are standard for perineal repair [17–19]. In 2004, Vicryl^®^ (Polyglactin 910, Ethicon), a commonly used suture, was introduced with a triclosan coating (VicrylPlus^®^). Triclosan, approved by the U.S. Food and Drug Administration, has been used in cosmetics and certain consumer products for over 30 years [20]. It inhibits microbial fatty acid synthesis, serving as a broad-spectrum bactericide with antibacterial and antifungal properties. In vivo and in vitro studies, as well as randomized controlled trials, have shown mixed results regarding the efficacy of antibacterial sutures in reducing surgical site infections (SSI). This ambiguity led to the recent publication of a Cochrane protocol by Wormald et al. to assess the effectiveness of antibacterial sutures [21–25]. Despite this, the National Institute for Health and Care Excellence (NICE) in the U.K. recommends antibacterial sutures such as triclosan-coated sutures (TCS) as the first-line choice for wound closure after most types of surgery [26].

This study aims to investigate the efficacy of TCS in preventing infection in a CRPT for up to 30 days after birth.

## Methods

*Outcome* The definition of infection was adapted from the Centers for Disease Control, Prevention (CDC), the World Union of Wound Healing Societies (WUWHS), and the REEDA scale, and was established by a healthcare professional based on the following criteria:

- Any one of the following criteria alone: (a) spontaneous dehiscence of tear (b) antibiotic prescribed due to tear-infection (c) abscess potentially with purulent or foul-smelling discharge in the tear.
- Or at least two of the following criteria: localized pain, edema/swelling, tenderness, redness of surrounding tissue, or heat [5, 27–29].

### Study design

The PLUS study was designed as a single-center; single-blinded, adaptive parallel-group randomized controlled trial at Skåne University Hospital, Lund. The birthing unit handled approximately 3,700 deliveries per year, including extremely premature births and newborns with severe conditions requiring specialized neonatal care and/or surgery. If a patient had a CRPT, it was graded (episiotomies were always mediolateral) by the attending midwife and, if necessary, by the coordinating midwife or the doctor on call. The midwives and doctors were familiar with the sutures, as they had been part of the unit’s standard assortment for several years. Eligible women were informed about the perineal tear and the necessity of suturing. A prepacked envelope containing sutures, a randomization number, a questionnaire to be completed at one and eight weeks postpartum (data and results not included in this article), an informed consent form, and the primary investigator’s (PI) phone number was selected by the midwife. If the woman agreed to participate, she signed the consent form, after which she was given the questionnaire and a stamped envelope. The documents (randomization number, patient identification, and signed consent form) were then placed in a locked box in the labor ward. If a woman declined to participate, the midwife unsealed the envelope to access the sutures, as they were the only ones available in the labor room.

### Participants

Eligible participants were women aged 18 years or older, with no language barriers, who had a perineal tear involving muscle and/or fascia that required suturing with a medium-lasting multifilament suture, regardless of intrapartum antibiotic use. Exclusion criteria included: tears only needing a fast-absorbable suture (e.g., VicrylRapid^®^) such as first degree-tears affecting the vagina mucosa or labia, tears requiring suturing in the operation theater due to guidelines (severe third- or fourth-degree tears) or complexity, history of elective perineal surgery, intrauterine fetal death or other serious health threatening conditions of the neonate (due to the physiological stressful situation for the parents), HIV or active Hepatitis B/C infection, and severe perineal warts or varicose veins.

### Procedure

Before the study began, midwives and doctors attended a mandatory course to standardize their expertise in diagnosing perineal tears and to suture with a continuous technique. All sutures except the 3 − 0 fast-absorbable polyglactin (VicrylRapid^®^) were removed from the labor rooms and replaced by the project-sealed envelopes. The perineum was always cleaned with water and non-sterile single-use towels, and an aseptic pad was placed under the woman’s buttocks before suturing. Sterile surgical instruments and gloves were used. The staff was instructed to suture the deeper layers and subcutis continuously, while the technique for skin adaptation was left to their discretion. Upon discharge, women received standard information on hygiene, signs of infection, pelvic floor function, and recommended postpartum exercises. Although no scheduled follow-up was provided, they were instructed to contact our post-partum ward, our gynecological emergency ward or the PI if they showed signs of infection. The emergency department, open 24/7, offered unrestricted access to examinations by the on-call doctor. Infections in a tear was determined exclusively in women who were either evaluated by the PI or presented themselves to our wards.

Relevant medical data were assessed from the electronic medical records: Obstetrics (Oracle Cerner^®^) and Melior (Siemens) which are the data systems used on all hospitals in the region.

### Randomization

The women were randomly allocated (1:1) to the intervention group (closure with the TCS, polyglactin VicrylPlus^®^) or the control group (closure with Vicryl^®^), in randomization blocks of 100, 50 in each group. Randomization blocks were created using the web-based program “Randomization.com.” The sutures were identical in all properties: purple-colored, braided thread, needle size CT-1, 1/2 circle. The randomization number was documented in the medical record without noting the suture type, which remained masked to healthcare providers (except the operator), the women, and the researchers until the database was completed.

### Statistical analysis

An adaptive group-sequential design was implemented to account for the uncertainty of the infection rate, allowing for recalculation of the sample size. The sample size calculation aimed to detect a 50% decrease in infection rates in the intervention group from an estimated prevalence of 10% [28]. A two-tailed test with a significance level of 5% (alpha 0.05) and a power of 80% resulted in a sample size of 960 women (480 in each arm). Results are presented as mean, standard deviation, median, IQR, number, percentage, and odds ratio/adjusted odds ratio with a 95% confidence interval. Statistical tests included unpaired t-tests for continuous variables, Fisher’s exact test for categorical variables, and binary logistic regression. *P* ≤ 0.05 was considered statistically significant. Statistical analyses were performed using the IBM Statistical Package for Social Sciences (SPSS) for Windows versions 27 and 28.

## Results

After one year, 838 women had been enrolled in the study. A second-degree tear (not differentiated between spontaneous or episiotomy, *n* = 544) was most common and the crude incidence of infection in the whole cohort was 5%. Based on this, a new power calculation determined that a sample size of 1,810 women (905 per group) was needed to demonstrate a 50% reduction in infection rates with an alpha of 0.05 and a power of 80%. Considering an estimated dropout rate of 5%, the total required sample size was 1,905 women (N1 = n / (1 - d), where d is the estimated dropout rate in percentage).

**Fig. 1:**
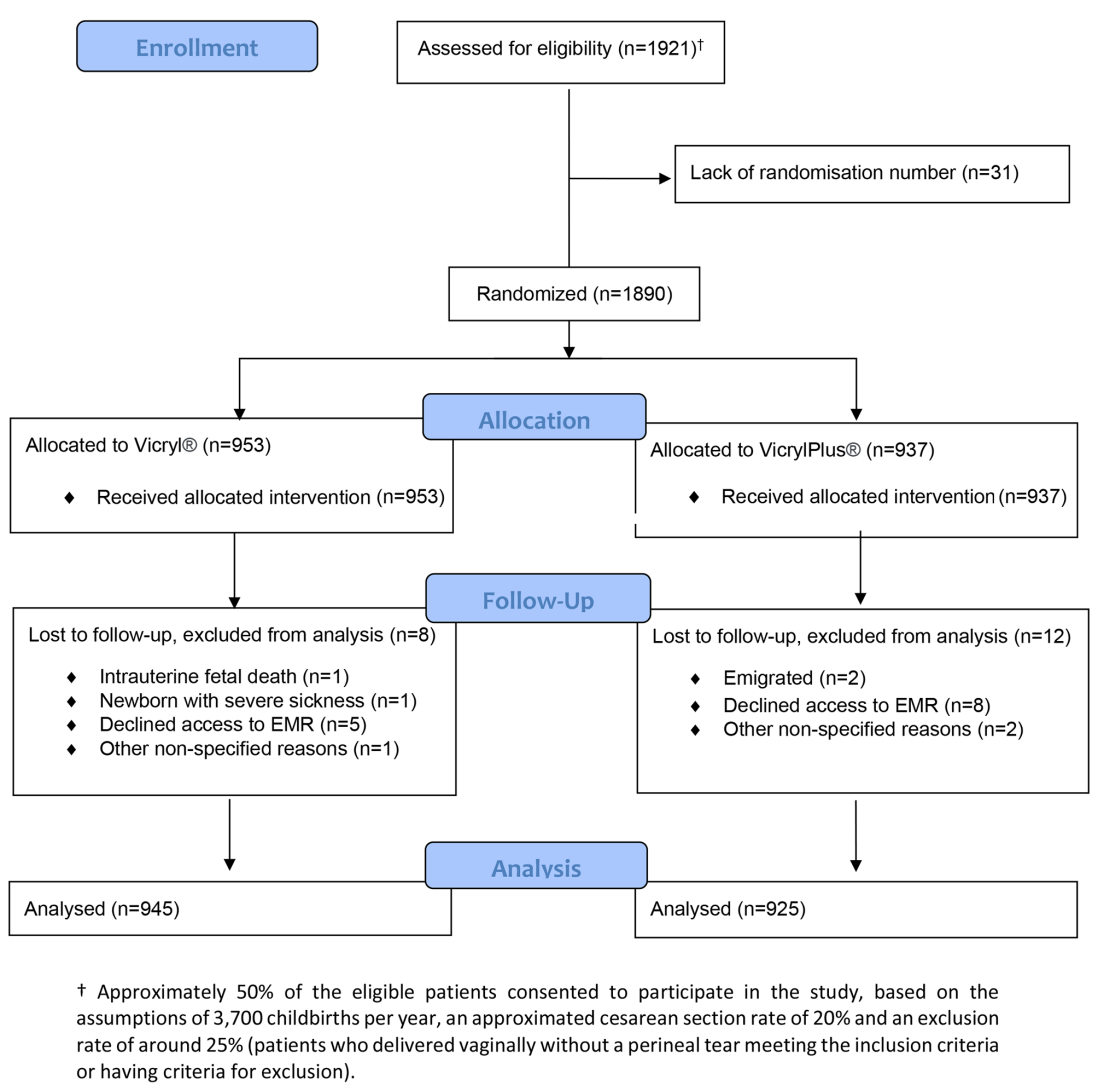
Trial profile. EMR: electronic medical record.

By February 2018, enrollment reached 1,921 women, at which point recruitment ceased. There was no report of side effects including allergic reaction to the sutures. Thirty-one women missed randomization numbers, leaving 1,890 participants for randomization into the intervention group (TCS, VicrylPlus^®^; *n* = 937) or the control group (non-coated suture, Vicryl^®^; *n* = 953). In the intervention and control groups, twenty women were lost to follow-up (12 women (1.2%) versus 8 women (0.8%)), resulting in 1,870 women available for final modified intention-to-treat analysis, Fig. 1. The primary reason for dropout was the withdrawal of informed consent. Among the participants, 583 women (63.5%) in the intervention group and 576 women (61.3%) in the control group were nulliparas, Table 1. The two study groups were equivalent, showing no significant differences in demographic and obstetric characteristics, maternal comorbidities, fetal outcomes, and antibiotic use, Table 1.

**Table 1:** Demographics of the study population

			Vicryl^®^ *N* = 945	VicrylPlus^®^ *N* = 925	*P* value
			Mean ± SD Median [IQR]	Mean ± SD Median [IQR]	
Maternal age, years			31.2 ± 4.6 31 [27.7; 34.3]	30.7 ± 4.5 30.4 [27.5; 33.8]	0.225^‡^
Maternal BMI			24.5 ± 4.3 23.8 [21.4; 26.8]	24.3 ± 4.1 23.5 [21.4; 26.4]	0.912^‡^
Gestational age, days			281 ± 9.1 281 [275; 287]	280 ± 9.9 281 [275; 287]	0.475^‡^
Fetal weight, grams			3636 ± 459 3613 [3346; 3920]	3614 ± 442 3612[3319; 3907]	0.938^‡^
Cord artery pH			7.23 ± 0.08 7.23 [7.18; 7.28]	7.23 ± 0.08 7.23 [7.17; 7.28]	0.455^‡^
			n (%)	n (%)	
Parity	Primipara Multipara		576 (61.3) 363 (38.7)	583 (63.5) 335 (36.5)	0.338*
Previous perineal tear (yes)			269 (32.3)	255 (31.0)	0.598*
Epidural anesthesia (yes)			263 (28.3)	245(26.7)	0.435*
Intrapartum antibiotic	None Narrow spectrum Broad spectrum		674 (73.1) 188 (20.4) 60 (6.5)	670 (74.1) 176 (19.5) 58 (6.4)	0.873*
Delivery mode	Partus normalis Instrumental vaginal delivery		853 (91.3) 81 (8.7)	831 (90.5) 87 (9.5)	0.572*
Tobacco during pregnancy (yes)			28 (3.1)	31 (3.4)	0.693*
Diabetes (yes)			4 (0.4)	5 (0.5)	0.751*
Genital herpes (yes)			10 (1.1)	12 (1.3)	0.673*
Connective tissue disease (yes)			10 (1.1)	10 (1.1)	1.000*
Education	Primary Secondary Tertiary		51 (5.8) 290 (32.8) 543 (61.4)	42 (4.8) 258 (29.6) 573 (65.6)	0.176*

SD: standard derivation, IQR: interquartile range, BMI; body mass index. ‡t-test for continuous variables, *Fisher’s exact test for categorical variables

The incidence of different tear types was similar in both groups. The most common were second-degree tears, with 510 cases (54%) in the control group and 518 cases (55.9%) in the intervention group, followed by first-degree tears and there after episiotomies. A CRPT was classified as ‘unclassified’ if the midwife or doctor had only described the tear in general terms, such as “4 cm long vaginal tear on the left side and 2 cm deep perineal tear” or “defect in the recto-vaginal fascia,” implying that it was always more than a first-degree tear (see Table 2).

**Table 2:** Distribution of perineal tears at delivery

		Vicryl^®^ *N* = 945	VicrylPlus^®^ *N* = 925	*P* value
		n (%)	n (%)	
First-degree tears		241 (25.5%)	210 (22.7%)	0.51*
Second-degree tears	Spontaneous Episiotomy	510 (54%) 115 (12.2%)	518 (55.9%) 107 (11.6%)	
Third-degree tears		14 (1.5%)	19 (2.1%)	
Unclassified		65 (7%)	72 (7.8%)	

*Fisher’s exact test

In the overall cohort, which included all types of CRPTs except type one tears—since they typically do not require suturing with an intermediate-lasting suture—the risk of infection was significantly lower in the intervention group: VicrylPlus^®^ 4% (*n* = 28) versus Vicryl^®^ 6.8% (*n* = 47) (OR 0.57, 95% CI 0.35–0.91, *P* = 0.024). In second-degree tears, VicrylPlus^®^ significantly reduced the risk of infection (OR 0.63, 95% CI 0.36–0.98) (Table 3). After adjusting for maternal age, body mass index, parity, antibiotic use, non-steroidal anti-inflammatory drug (NSAID) use in the first week postpartum, GBS infection, and mode of birth, only minor changes in the odds ratios were observed. In the crude cohort, several factors demonstrated significant associations with an increased risk of infection: nulliparous women (OR 2.95 (95% CI 1.67–5.19), *P* < 0.001), NSAID use during the first week post-partum (OR 2.5 (95% CI 1.5–4.27), *P* < 0.001), episiotomy compared to spontaneous second-degree tear (OR 4.58 (95% CI 2.80–7.5), *P* < 0.001) and assisted vaginal birth (OR 4.36 (95% CI 2.64–7.21), *P* < 0.001). Conversely, having a prior perineal tear significantly reduced the risk (0.3 (95% CI 0.15–0.58), *P* < 0.001). A body mass index of 30 or higher was not associated with an increased risk of infection (OR 1.11, 95% CI 0.54–2.27, *P* = 0.850), Table not shown. If all the aforementioned factors were included in a binary logistic regression model, NSAID use, episiotomy, and assisted vaginal birth remained significantly associated with a higher risk for infection, Table not shown.

**Table 3:** Primary outcome: infection in childbirth related perineal tears (CRPT) up to one month after delivery

	Vicryl^®^ exposed/total cases (%)	VicrylPlus^®^ exposed/total cases (%)	*P* value	OR (95% CI)	Adj- OR (95% CI)
Overall infection rate in CRPT except grade one tears	47/687 (6.8%)	28/702 (4%)	0.024^*^	0.57 (0.35–0.91)	0.55 (0.31-1.00)
First-degree tears	2/236 (0.8%)	8/205 (3.9%)	0.050^*^	4.75 (1.00-22.63)	NA
Second-degree tears (spontaneous and episiotomies)	44/613 (7.2%)	27/613 (4.4%)	0.050^*^	0.60 (0.36–0.98)	0.63 (0.34–1.15)
Spontaneous second-degree tears Episiotomies	24/502 (4.8%)	14/507 (2.8%)	0.100^*^	0.57 (0.29–1.11)	0.73 (0.33–1.60)
	20/111 (18%)	13/106 (12.3%)	0.261^*^	0.64 (0.30–1.35)	0.56 (0.20–1.62)
Third-degree tears	2/14 (14.3%)	1/19 (5.3%)	0.561^*^	0.33 (0.03–4.10)	NA
Unclassified (except grade one CBPT)	1/60 (1.7%)	0/70 (0%)	0.462^*^	0.98 (0.95–1.02)	NA

^*^Fisher’s exact test. Adjusted for maternal age, maternal body mass index, primipara, antibiotic, non-steroid-anti-inflammatory-drug, GBS infection, and delivery mode

## Discussion

This large randomized, single-center clinical trial demonstrated that the use of triclosan-coated sutures significantly reduces the risk of infection in a primary sutured CRPT with exception of first-degree tears. This finding is particularly important as second-degree tears are the most frequent tears encountered during childbirth [9, 10].

To the best of our knowledge, this is the only randomized controlled trial investigating the efficacy of TCS for the primary suturing of CRPT. Initial studies on TCS demonstrated promising results in both in-vitro and in-vivo settings, however, subsequent human studies have reported mixed findings [23, 25, 30–35]. The trial led by Renko et al. (clinics in Finland, different types of pediatric surgery) and the trial led by Mbarki et al. (clinics in Tunisia, cesarean section) found a substantial and significant reduction in SSIs (RR 0.48, 95% CI 0.28–0.80; respective ORa 0.294, 95% CI 0.094–0.921) [32, 36]. In contrast the FALCON trial (different surgery across low- and middle-income countries) and the PROUD trial (clinics in Germany, abdominal wall-closure) did not show any significant benefit from TCS (clean wound RR 0.90 (95% CI 0.77–1.06), dirty wound RR 0.98 (95% CI 0.87–1.10); respective OR 0.91, 95% CI 0.66–1.25; *P* = 0.64) [33, 34]. The authors of the PROUD trial included a meta-analysis on TCS exclusively for abdominal wall closure, which revealed a significant benefit of the antibacterial sutures (OR 0.67, 95% CI 0.47–0.98). They suggested that differences in statistical power and single-center versus multi-center study designs might explain discrepancies between their findings and those of previous meta-analyses [34]. Another recent comprehensive meta-analysis including 11,957 patients across 25 randomized controlled trials supports the use of TCS in reducing the risk of SSIs (RR 0.73, 95% CI 0.65 to 0.82) [37]. The analysis are based on a wide range of surgeries, low and high-income countries and in both adult and pediatric patients.

In cases of first-degree perineal tears, our study observed an increased, though not statistically significant, risk of infection associated with the use TCS. This finding may be attributed to the small sample size but could also relate to the unique biological environment of the vaginal area. The vagina has a highly effective “self-cleaning” mechanism, characterized by natural secretions and a stable pH that supports its defense against infections. We hypothesize that the prolonged presence of suture material in this environment might disrupt these natural processes, potentially impairing the vagina’s innate defense mechanisms and altering its microbiota or pH balance, as suggested by prior studies on vaginal health and tear healing [19, 38, 39]. Our findings also indicate that TCS are significantly more effective in more profound tears, corroborating results from Renko et al., who reported an approximately 80% reduction in SSI rates for deep tears compared to a 40% reduction in superficial tears (the latter being non-significant) [36]. This aligns with broader evidence suggesting that TCS are particularly beneficial in reducing SSIs in surgeries involving deeper tissue layers, possibly due to their sustained antimicrobial activity in environments with higher bacterial loads [35, 36]. A recommendation of the use of TCS in primary perineal repair will align with recommendations in the NICE guidelines that suggest that while confidence in the benefits of TCS is limited, the suture should still be considered for wound closure in all types of surgery until further research clarifies their optimal use [26].

There is a lack of consensus in the literature regarding the definition of an infection in a CRPT, which complicates comparisons of incidence across studies and according to the Centers for Disease Control and Prevention (CDC), standard criteria for surgical site infections only apply to episiotomies [27]. A systematic review found that the reported incidence of perineal wound infections ranges from 0.1 to 23.6%, reflecting differences in study designs, settings, and diagnostic criteria [40]. Scandinavian countries, with relatively homogenous populations and consistent obstetrical practices, have conducted several studies on the incidence of perineal wound infections [41–44]. A recent Swedish study on assisted vaginal births reported infection rates of 5% in spontaneous wounds and 9% in episiotomies [44]. The study relied on data from electronic medical records and did not clearly define perineal wound infection. In contrast, a Danish study that assessed all patients postpartum in a clinical setting reported infection rates of 9% in spontaneous second-degree tears and 11% in episiotomies and if the patient had received prophylactic antibiotics due to a sphincter injury, the infection rate was lowered to 3%. In the study, infections were defined as purulent discharge or abscess formation with wound dehiscence of 0.5 cm or more [42]. In our study, the rate of infection in episiotomies was higher (18% in the Vicryl^®^ group versus 12.3% in the VicrylPlus^®^ group). Whether these findings can be explained by differences in the definition of infection or variations in clinical practices that affects the infection risk remains unclear. Potential the use of continuous suturing techniques and the anatomical characteristics of medio-lateral episiotomies can contribute to the difference. In our study the staff was trained and instructed to suture continuously and while continuous suturing offers advantages in many contexts, it can be challenging to execute effectively in episiotomies, potentially leading to inadequate wound approximation and increased bacterial access [45]. It is also important to mention the anatomical differences between spontaneous second-degree tears and medio-lateral episiotomies. The subcutaneous tissue around a medio-lateral episiotomy is thicker compared to the region between the vaginal inlet and anus, and differences in moisture and temperature between these areas may influence the natural bacterial flora. Furthermore, the intentional incision of an episiotomy may disrupt blood supply differently than a spontaneous tear, potentially affecting healing and infection risk. This underscores the importance of adopting a restrictive approach to episiotomy use [45]. Although evidence remains insufficient to recommend routine prophylactic antibiotics for episiotomies, we agree with existing guidelines suggesting that antibiotics should be considered when episiotomies are combined with other risk factors for infection, such as complex tears or instrumental assisted births [46].

Concerns have been raised about the potential impact of triclosan and its dioxin-like degradation products on both human health and the environment [47, 48]. The removal of triclosan from wastewater remains a challenge due to its resistance to biodegradation [49]. These environmental concerns should lead to restricting TCS to applications where benefits are well-documented. On the other hand, the use of TCS may have a comparatively lower environmental impact than systemic antibiotics [50, 51].

Our study offers several strengths: it potential included all women who gave birth in the birthing unit and required sutures with intermediate absorption time, as only the pre-packaged study envelopes were available in the labor room. Prior to initiation, all doctors and midwives performing repairs received training to ensure high-quality and homogeneous practice. If the type of tear or structures involved were not clearly defined, the wound was analyzed as “unclassified” to secure high data quality. In accordance with the study protocol, the midwives performing the repair were not formally blinded to the type of suture. However, we do not consider this a significant limitation, as the attending midwife did not encounter the woman after the she was discharged from the birthing unit. The measures taken in our study to maintain masking, combined with the standard practices in the labor ward, effectively minimized the potential for bias in suture application and subsequent tear assessment.

Women were not offered scheduled follow-ups in this study, but the diagnosis of CRPT infection was always made by a doctor. Generally, all women with potential infections in a CRPT are referred to the hospital for diagnosis and electronic medical records were accessed for all enrolled patients, ensuring insignificant risk that a woman with infection was lost to follow-up. We calculated the statistical power based on the most recent and suitable data available at the time. After the interim analysis, we found that the incidence of infection in a CRPT was approximately 5%, and the study population was recalculated accordingly. Sphincter injuries were generally not included, as these women always received broad-spectrum antibiotics and logistical challenges made securing randomization difficult. Therefore, for CRPTs other than second-degree tears (both spontaneous and episiotomies), the statistical power was too low to draw definitive conclusions. Further studies with larger populations are needed to assess the effectiveness in the other types of CRPTs (three and four-degree tears).

It is important to note that our study population primarily consisted of Swedish-born patients living in an area with generally good socioeconomic resources, which may limit the generalizability of our results to other populations. Although data collection was delayed due to the time-consuming nature of electronic medical record systems, we do not consider this a significant limitation, as the methods and materials used remain relevant, and the retrieved data was not altered.

## Conclusions

Infection in childbirth-related perineal tears affects approximately 5% of women. The PLUS study demonstrated that TCS effectively reduces infection rates by nearly 50% in primary sutured CRPTs, excluding first-degree tears. These findings have important clinical implications, supporting the use of antibacterial sutures for closing deeper perineal tears that involve subcutaneous tissue, fascia, and muscle. Further research in a larger study is needed to determine whether their effectiveness remains consistent across all types of perineal tears.

## Electronic supplementary material

Below is the link to the electronic supplementary material.

Supplementary Material 1

## Data Availability

No datasets were generated or analysed during the current study.
